# A deep learning-based multi-modal fusion framework for football foul recognition and referee decision assistance

**DOI:** 10.1038/s41598-026-58030-y

**Published:** 2026-06-23

**Authors:** Chunxiang Xue, Zheng Gao

**Affiliations:** Department of Sports and Leisure, Dongshin University, Naju, 58245 South Korea

**Keywords:** Deep learning, Football foul recognition, Multi-modal fusion, FoulMFNet, Attention mechanism, Referee decision assistance, Action recognition, Engineering, Mathematics and computing

## Abstract

Accurate referee decision-making remains a critical challenge in football, where the distinction between legal and illegal contact often depends on subtle contextual factors. This paper presents FoulMFNet, an integrated deep learning framework for automated foul recognition designed to assist officiating judgment. The proposed system employs a multi-modal fusion architecture that jointly processes appearance features, motion dynamics, and skeletal representations through dynamically weighted integration. An attention-based temporal focusing mechanism identifies decisive contact moments within extended video sequences, while a confidence calibration module produces well-calibrated probability estimates reflecting prediction reliability. Comprehensive experiments conducted on a dataset comprising 12,463 incident clips from professional matches demonstrate that the proposed framework achieves 87.3% recognition accuracy across five foul categories, outperforming established baseline methods by 4.1 to 10.4 percentage points. Consistency analysis with FIFA-certified referees yields a Cohen’s kappa of 0.82, indicating substantial agreement with professional standards. The system maintains real-time performance with median latency of 127 ms, enabling practical deployment alongside existing Video Assistant Referee infrastructure. Interpretability components provide comprehensible visualizations of decision rationale, facilitating referee evaluation of system recommendations.

## Introduction

Football remains one of the most widely followed sports globally, with billions of viewers tuning in to major tournaments each year. The accuracy of referee decisions has long been a contentious issue that directly influences match outcomes and, by extension, the credibility of competitive sports^1^. Traditional refereeing relies heavily on human observation, which inevitably suffers from limitations such as restricted viewing angles, cognitive overload, and the sheer speed at which critical incidents unfold on the pitch^2^. These inherent constraints have prompted growing interest in technological interventions that might augment human judgment rather than replace it entirely.

Over the past decade, researchers have explored various approaches to automated sports analysis. Early attempts focused primarily on player tracking and event detection using conventional image processing techniques^3^. More recently, the convergence of computer vision and deep learning has opened new possibilities for understanding complex game scenarios^4^. Convolutional neural networks, in particular, have demonstrated remarkable capabilities in extracting spatial features from video streams, enabling more nuanced recognition of player movements and physical contacts^5^. Several studies have investigated pose estimation methods for analyzing player interactions during potential foul situations^6^. Meanwhile, recurrent architectures have been employed to capture temporal dependencies inherent in dynamic sports sequences^7^.

Despite these advances, significant challenges persist. Foul recognition differs fundamentally from other action recognition tasks because the distinction between legal and illegal contact often hinges on subtle contextual factors—intent, force magnitude, and the specific rules governing different types of challenges^8^. Current systems struggle to generalize across diverse match conditions, camera setups, and playing styles prevalent in different leagues^9^. The scarcity of annotated datasets containing verified foul incidents further compounds these difficulties, as supervised learning approaches require substantial labeled examples to achieve robust performance^10^.

The practical significance of developing reliable referee assistance systems extends beyond mere technological novelty. Incorrect penalty decisions can alter championship trajectories and generate substantial controversy, undermining public trust in sporting institutions^11^. A well-designed decision support framework could provide referees with real-time supplementary information, helping them make more informed judgments without disrupting the natural flow of the game.

This paper presents an integrated approach combining multi-scale feature extraction with attention mechanisms specifically tailored for foul recognition scenarios. The proposed system introduces a novel temporal-spatial fusion module that captures both instantaneous contact characteristics and preceding motion patterns. Furthermore, we develop a confidence calibration mechanism that quantifies decision uncertainty, thereby enabling referees to prioritize cases warranting closer review^12^. Through extensive experiments on both benchmark datasets and newly collected match footage, we demonstrate that the proposed framework achieves superior recognition accuracy while maintaining computational efficiency suitable for real-time deployment.

## Theoretical foundations and technical basis for football foul recognition

### Applications of computer vision in sports scenarios

Computer vision fundamentally seeks to enable machines to interpret and understand visual information from the physical world. At its core, the discipline transforms raw pixel data into meaningful representations through hierarchical feature extraction processes^13^. Object detection, arguably the most critical component for sports analysis, aims to localize and classify multiple entities within a single frame. Modern detection frameworks typically formulate this task as a regression problem, where bounding box coordinates and class probabilities are predicted simultaneously. The Intersection over Union (IoU) metric quantifies localization quality by computing the ratio of the overlap area to the union area between a predicted bounding box and its corresponding ground-truth box. Building on this metric, the standard detection confidence score combines classification confidence with localization quality:

1 $$S\left( {\det } \right) = P(class|object) \times IoU_{pred}^{truth}$$

where P(class|object) denotes the conditional class probability given an object is present, and $Io{U}_{pred}^{truth}$ measures spatial agreement between the predicted and ground-truth bounding boxes. It bears noting that this expression is not a formal probability in the strict probabilistic sense; rather, it serves as a composite confidence score that jointly reflects how well the detector classifies and localizes objects. This scoring formulation, widely adopted in detection architectures, balances classification accuracy against localization precision.

Tracking moving objects across video sequences presents additional complexity. The Kalman filter remains a foundational approach for predicting object trajectories under uncertainty^14^. Given a state vector ${x}_{t}$ representing the position and velocity of a tracked object at frame t, the prediction step follows:

2 $$x_{t|t - 1} = Fx_{t - 1} + w_{t}$$

where ${x}_{t-1}$ is the state estimate at the previous frame, F denotes the state transition matrix that models expected motion between consecutive frames, ${x}_{\left(t|t-1\right)}$ is the predicted state for frame t given observations up to frame t − 1, and ${w}_{t}$ represents process noise accounting for deviations from the assumed motion model. Sports scenarios, however, impose unique demands on tracking algorithms—rapid direction changes, frequent occlusions, and visually similar players wearing identical jerseys all complicate correspondence across frames^15^.

What makes sports video analysis particularly challenging? The answer lies partly in the dynamic nature of athletic motion. Player velocities can exceed 30 km/h during sprints, resulting in significant motion blur at standard broadcast frame rates. Camera movements—panning, zooming, and cutting between views—introduce additional variability that static scene algorithms simply cannot handle. The affinity between detected objects across frames is often computed using appearance and motion cues:

3 $$A_{i,j} = \alpha \cdot sim\left( {f_{i} ,f_{j} } \right) + \left( {1 - \alpha } \right) \cdot IoU\left( {b_{i} ,b_{j} } \right)$$

Here, ${A}_{i,j}$ represents the affinity score between detection i in one frame and detection j in a subsequent frame. The terms ${f}_{i}$ and ${f}_{j}$ are the corresponding appearance feature vectors extracted from each detection, ${b}_{i}$ and ${b}_{j}$ denote their respective bounding boxes, sim(·) measures cosine similarity between appearance features, IoU(·) captures spatial overlap between bounding boxes, and α ∈ [0,1] is a weighting coefficient that controls the relative importance of appearance versus spatial information^16^.

Recent progress in end-to-end learning has substantially advanced sports video understanding. Transformer-based architectures now achieve state-of-the-art performance on action spotting benchmarks, outperforming earlier convolutional approaches by considerable margins^17^. These developments provide essential building blocks for the foul recognition system proposed in this study.

### Deep learning models and action recognition technology

Convolutional neural networks have revolutionized visual feature extraction by learning hierarchical representations directly from raw imagery. A standard convolutional operation applies learnable filters across spatial dimensions, generating feature maps that capture increasingly abstract patterns at deeper layers^18^. The convolution for a given input *X* and kernel *W* follows:

4 $$Y_{i,j} = \mathop \sum \limits_{m}^{{}} \mathop \sum \limits_{n}^{{}} X_{i + m,j + n} \cdot W_{m,n} + b$$

where X is the input feature map, ${W}_{\left(m,n\right)}$ represents the learnable kernel weights indexed by spatial offsets m and n, and b is the bias term. This operation is fundamental to all CNN architectures and produces translation-equivariant feature maps—meaning that when an input pattern shifts in spatial position, the resulting feature map shifts correspondingly, preserving the spatial relationship. It is through subsequent pooling layers that approximate translation invariance emerges, enabling the network to recognize objects across varying positions within frames.

Yet spatial features alone prove insufficient for understanding actions, which inherently unfold across time. Recurrent neural networks address this limitation by maintaining hidden states that encode temporal context. The Long Short-Term Memory variant introduces gating mechanisms to mitigate vanishing gradient problems during backpropagation through extended sequences^19^. The forget gate, a critical component, determines which information to discard:

5 $$f_{t} = \sigma \left( {W_{f} \cdot \left[ {h_{t - 1} ,x_{t} } \right] + b_{f} } \right)$$

where *σ* denotes the sigmoid activation and ${h}_{t-1}$ represents the previous hidden state.

Attention mechanisms have emerged as perhaps the most influential innovation in recent deep learning research. Rather than treating all input elements equally, attention computes weighted combinations based on relevance scores^20^. The scaled dot-product attention, now standard in transformer architectures, calculates:

6 $$Attention\left( {Q,K,V} \right) = softmax\left( {\frac{{QK^{T} }}{{\sqrt[{}]{{d_{k} }}}}} \right)V$$

The scaling factor $\sqrt[{}]{{d_{k} }}$ prevents dot products from growing excessively large, which would push softmax outputs toward extreme values.

For action recognition specifically, two-stream architectures that process appearance and optical flow separately have demonstrated strong performance^21^. Pose estimation offers a complementary approach—by extracting skeletal keypoints, these methods reduce sensitivity to background clutter and clothing variations^22^. Graph convolutional networks operating on skeleton data now achieve competitive results while requiring substantially fewer parameters than dense video models.

Spatiotemporal feature extraction remains an active research frontier. Three-dimensional convolutions extend the standard 2D operation to jointly capture spatial and temporal patterns, though computational costs increase considerably^23^. Factorized approaches decompose 3D convolutions into separate spatial and temporal components, achieving efficiency gains without sacrificing recognition accuracy. These architectural innovations collectively provide the technical foundation upon which our foul recognition framework builds.

### Football foul behavior analysis and penalty rules

Football regulations distinguish several categories of fouls, each carrying different sanctions depending on severity and context. Direct free kick offenses include actions such as kicking, tripping, pushing, and striking opponents—violations that referees must identify through rapid visual assessment during continuous play^24^. The challenge lies in distinguishing legitimate challenges from illegal contact, a distinction that often hinges on timing, force, and whether the player genuinely attempted to win the ball.

From a biomechanical perspective, foul actions are associated with certain kinematic characteristics, though we must be candid about what broadcast video can and cannot reveal. The underlying physics of tackle impact follows the impulse-momentum relationship: $F_{impact} = \frac{\Delta p}{{\Delta t}} = \frac{{m\left( {v^{2} - v^{1} } \right)}}{\Delta t}$, where m is the player’s mass, v₁ and v₂ are velocities before and after contact, and Δt is the contact duration. We note, however, that precise values of impact force and contact duration cannot be reliably extracted from standard broadcast footage; this relationship instead offers conceptual motivation for why high relative velocity and abrupt contact tend to correlate with more severe fouls^25^.

Similarly, the angular velocity of leg movements during sliding tackles—expressed as ω = dθ/dt, where θ denotes joint angle—provides a theoretical basis for distinguishing reckless from controlled challenges. Excessive rotational motion, particularly when studs are raised, signals dangerous play. Again, accurate measurement of angular velocity from broadcast camera angles remains impractical; the relationship serves as a guiding principle rather than a directly computed feature.

Building on these biomechanical intuitions, we conceptualize a contact severity index that incorporates spatial and kinematic factors: $CSI = \alpha \cdot \left| {v_{rel} } \right| + \beta \cdot \theta_{leg} + \gamma \cdot d^{ - 1}$, where $v_{rel}$ represents the relative velocity between players, $\theta_{leg}$ denotes leg elevation angle, d measures player separation at contact initiation, and α, β, γ are weighting coefficients. Rather than computing this index directly from video, the formulation captures the physical reasoning behind our feature design choices: the model learns proxies for these quantities through appearance, optical flow, and pose features that indirectly reflect the kinematic conditions associated with fouls of varying severity.

Video Assistant Referee technology has transformed officiating in elite competitions, yet its implementation reveals persistent shortcomings^26^. VAR reviews remain human-dependent, subjective interpretations still vary, and not all leagues possess the infrastructure for comprehensive coverage. Processing delays disrupt match flow, sometimes by several minutes. Perhaps most problematically, VAR addresses only clear and obvious errors—borderline incidents, which constitute the majority of controversial calls, frequently escape correction^27^. These limitations underscore the need for automated analytical tools that can provide objective, consistent assessments to support rather than replace human judgment.

## Design of deep learning-based football foul recognition system

### Overall system architecture design

The proposed referee decision assistance system adopts a modular pipeline architecture that processes broadcast video streams through sequential yet interconnected stages. We designed this framework with two primary objectives in mind: achieving real-time performance suitable for match deployment while maintaining sufficient analytical depth to capture subtle foul indicators. The architecture balances computational efficiency against recognition accuracy, a tradeoff that pervades all practical computer vision applications^28^.

**Fig. 1:**
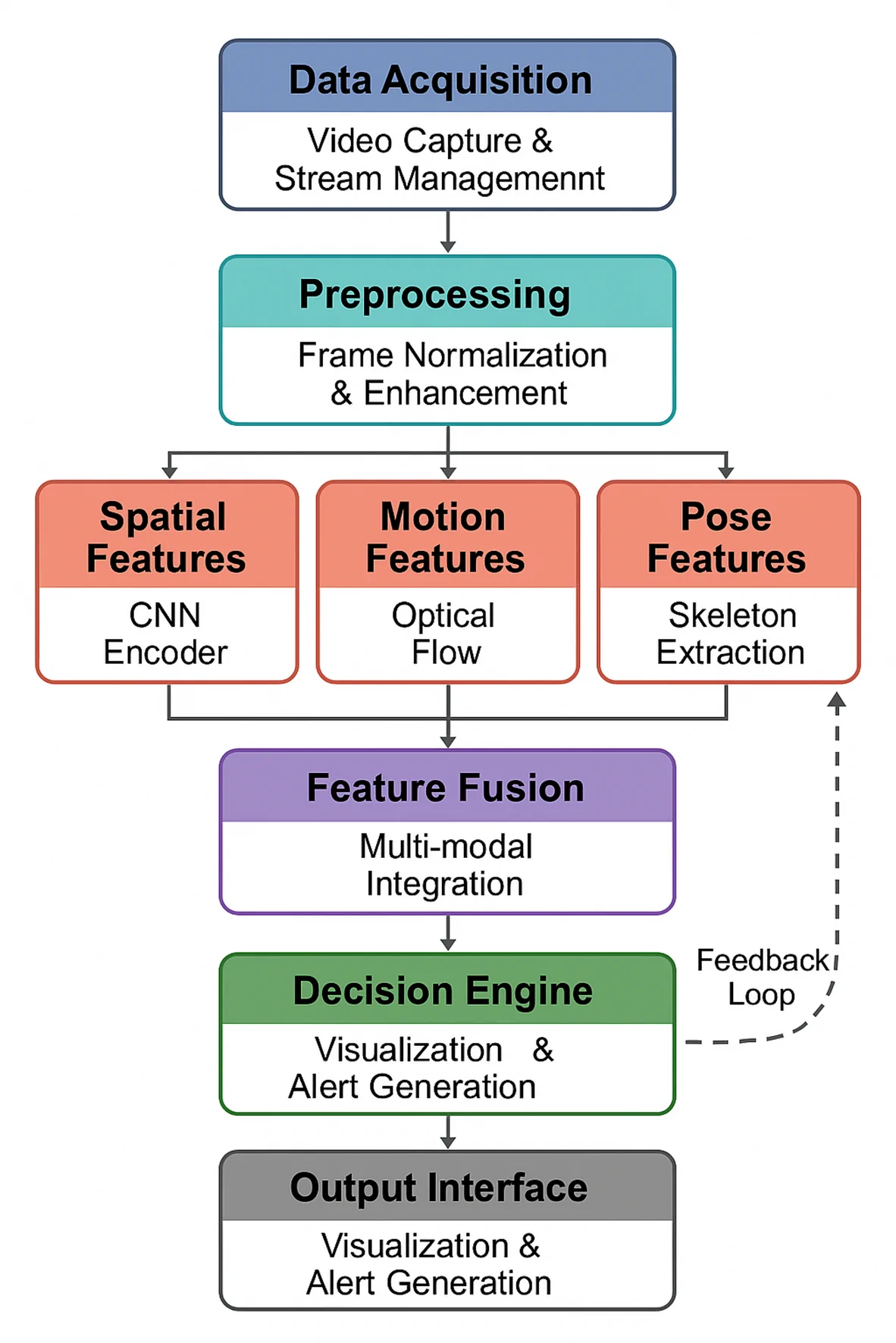
Overall Architecture of the FoulMFNet System. Note: In the revised figure, (**a**) the typographic error has been corrected, (**b**) color coding is unified across all modules with a consistent palette, and (**c**) the description boxes for “Decision Engine” and “Output Interface” have been revised to clearly differentiate their roles.

Figure 1 illustrates the complete system workflow, encompassing data ingestion through final decision output. The framework operates through five principal modules that communicate via standardized interfaces, enabling independent optimization of each component without disrupting the overall pipeline.

The data acquisition and preprocessing module serves as the system’s entry point. Raw video feeds undergo resolution normalization, frame rate standardization, and temporal segmentation into analyzable clips. Color space transformations and contrast enhancement address varying broadcast conditions across different stadiums and lighting environments^29^. This preprocessing stage proves critical—inconsistent input quality propagates errors throughout downstream modules.

Feature extraction constitutes the computational core of our system. This module deploys parallel processing streams: one dedicated to spatial appearance features through convolutional encoders, another focused on motion patterns via optical flow computation. A third stream performs real-time pose estimation for all visible players, generating skeletal representations that abstract away visual complexity. These heterogeneous feature types subsequently merge through learned fusion layers^30^.

The decision module receives concatenated feature representations and produces foul probability estimates alongside severity classifications. Rather than simple binary outputs, this component generates confidence scores that quantify prediction uncertainty. Such calibrated outputs enable referees to prioritize which incidents warrant closer examination. A temporal consistency mechanism smooths predictions across adjacent frames, reducing spurious detections caused by momentary occlusions or motion blur.

Table 1 summarizes the functional responsibilities and technical specifications of each system component. As presented in Table 1, the modular design facilitates incremental upgrades—individual modules can be replaced with improved implementations as new techniques emerge.

**Table 1:** System function module description.

Module name	Primary FUNCTION	Key technologies
Data acquisition	Video capture, stream synchronization, and feed management from multiple broadcast sources	Multi-camera synchronization, RTMP streaming protocol
Preprocessing	Frame normalization, resolution standardization, and visual quality enhancement	Adaptive histogram equalization, temporal sampling, color space transformation
Feature extraction	Multi-modal representation learning across appearance, motion, and pose streams	Modified ResNet-50 backbone, FlowNet 2.0, HRNet-W32 pose estimator
Decision engine	Foul category classification, severity scoring, and confidence calibration of predictions	Transformer decoder, temperature-scaled calibration, ensemble disagreement detection
Output interface	Visualization of interpretability outputs and prioritized alert delivery to referee operators	Grad-CAM heatmap rendering, temporal attention timeline display, confidence-ranked alert queuing
Feedback loop	Continuous model refinement through expert corrections accumulated during deployment	Active learning selection, incremental fine-tuning

The feedback mechanism deserves particular emphasis. Expert referees can annotate system outputs, and these corrections flow back into training data repositories^31^. This closed-loop design enables continuous model improvement through deployment experience, addressing the domain shift challenges that often degrade performance when systems transfer from training to operational environments.

### Foul behavior recognition model construction

Recognizing foul actions demands a model architecture capable of capturing both fine-grained spatial details and extended temporal dependencies. Standard video classification networks often struggle with this dual requirement—they either emphasize appearance at the expense of motion dynamics or vice versa. Our proposed architecture addresses this tension through a multi-scale feature pyramid that processes spatial information at varying resolutions while maintaining temporal coherence across the sequence^32^.

**Fig. 2:**
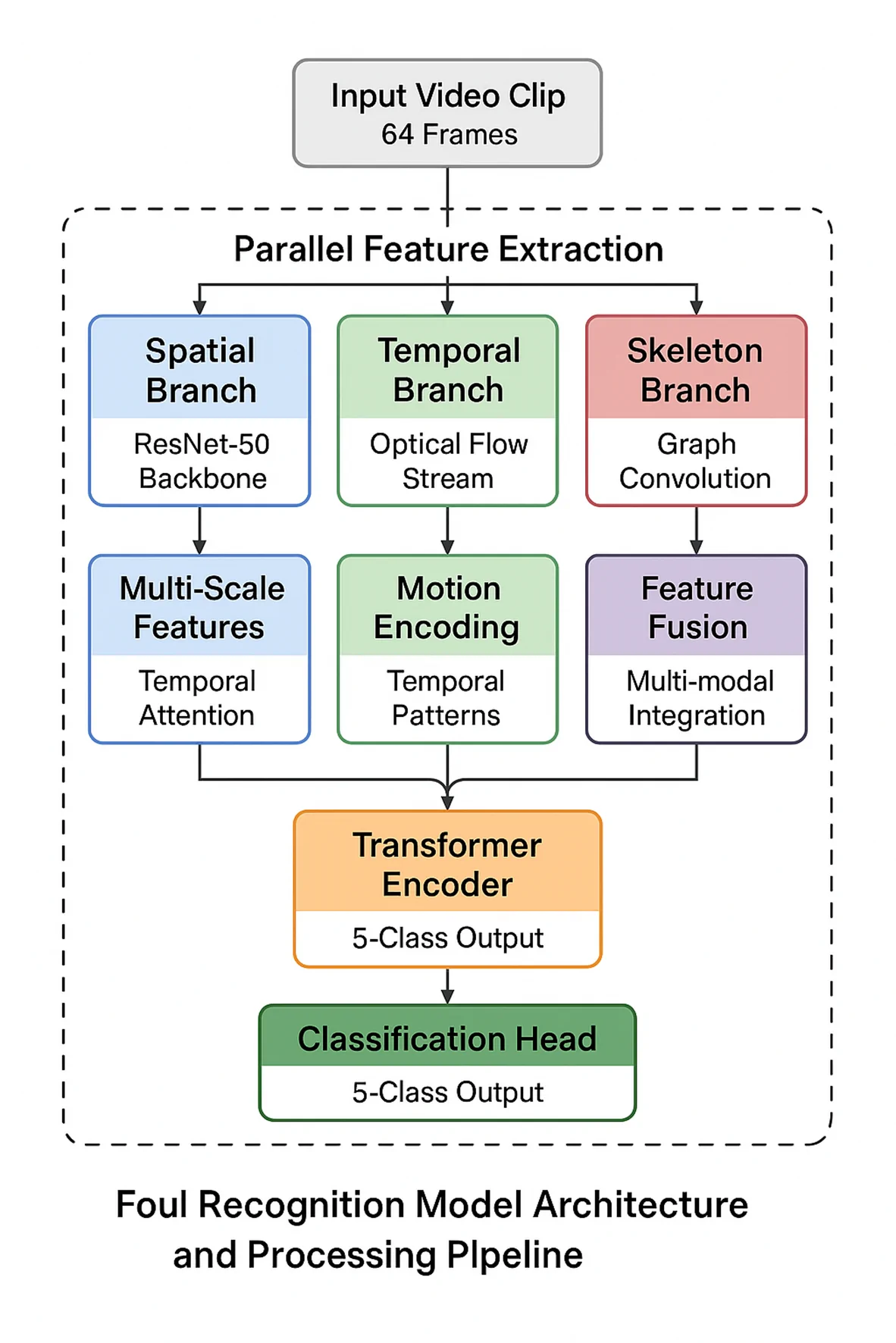
Foul recognition model architecture and processing pipeline.

Figure 2 depicts the complete model pipeline from input video clips to foul classification outputs. The architecture comprises three interconnected branches that converge at a fusion layer before final classification.

We refer to the proposed framework as FoulMFNet (Foul Multi-modal Fusion Network) throughout the remainder of this paper. The architecture comprises three specialized branches that process complementary information streams before converging at a learned fusion layer.

The appearance branch extracts hierarchical spatial features through progressively deeper convolutional stages. We adopt a ResNet-50 backbone^5^ pre-trained on ImageNet, with two key modifications: (a) we replace standard convolutions in Stage 4 with dilated convolutions (dilation rate = 2) to preserve spatial resolution at 1/16 of the input while expanding the effective receptive field, and (b) we attach Feature Pyramid Network lateral connections^32^ to fuse multi-scale feature maps from Stages 2 through 4. This design preserves both local texture detail and broader contextual information that proves essential for distinguishing contact types. The multi-scale feature aggregation follows:

7 $$F_{fused} = \mathop \sum \limits_{i = 1}^{L} w_{i} \cdot \phi_{i} \left( {F_{i} } \right)$$

where ${F}_{i}$ denotes features from scale *i*, ${\phi}_{i}$ represents learned projection functions, and ${w}_{i}$ indicates learnable fusion weights.

Not all frames contribute equally to foul identification. The moment of contact typically spans only a few frames within longer video segments. Our attention-based key frame selector dynamically weights temporal positions based on their discriminative value^33^. The temporal attention score computation proceeds as:

8 $$\alpha_{t} = \frac{{\exp \left( {f_{attn} \left( {h_{t} } \right)} \right)}}{{\mathop \sum \nolimits_{j = 1}^{T} \exp \left( {f_{attn} \left( {h_{j} } \right)} \right)}}$$

This mechanism effectively learns to focus computational resources on critical moments while suppressing irrelevant background frames.

The motion branch processes pre-computed optical flow fields generated by FlowNet 2.0^51^ with default parameters. We extract optical flow between consecutive frames at the native input frame rate, producing two-channel (horizontal and vertical displacement) flow maps. These flow maps feed into a separate ResNet-18 encoder, lighter than the appearance backbone, which captures motion magnitude and direction patterns characteristic of different foul types. Optical flow computation adds approximately 15 ms per frame on our deployment hardware.

The pose branch deploys HRNet-W32^22^ to estimate 17 COCO-format body keypoints per detected player. We retain only keypoint detections exceeding a confidence threshold of 0.4 to suppress unreliable estimates, a value determined through validation set tuning. For each incident clip, we extract skeletal representations of the two players closest to the estimated contact point, generating paired skeleton sequences that encode body configuration and inter-player spatial relationships^34^. The relative positioning between interacting players proves particularly informative—joint distances, limb angles, and velocity vectors all contribute discriminative signals. These skeleton features then undergo graph convolution to capture anatomical connectivity: $H^{{\left( {l{ + }1} \right)}} = \sigma \left( {\tilde{A}H^{\left( l \right)} W^{\left( l \right)} } \right)$, where $H^{\left( l \right)} \in {\mathbb{R}}^{{N \times d_{l} }}$ is the node feature matrix at layer *l* with *N = 17* keypoints and $d_{l}$ feature channels, $\tilde{A}$ is the normalized adjacency matrix encoding skeletal connectivity (with added self-loops), $W^{\left( l \right)} \in {\mathbb{R}}^{{d_{l} \times d_{l + 1} }}$ contains learnable projection parameters at layer *l*, and *σ* denotes the ReLU activation function. We stack three such graph convolutional layers, progressively mapping from raw keypoint coordinates to a 256-dimensional skeleton feature vector.

Table 2 lists the detailed layer configurations and parameter counts for the primary network components. As presented in Table 2, the total parameter count remains manageable for real-time inference on modern GPU hardware.

**Table 2:** Network layer parameter configuration.

Layer name	Input dimension	Output dimension	Parameters (M)
Conv1 block	3 × 224 × 224	64 × 112 × 112	0.12
ResBlock Stage2	64 × 112 × 112	256 × 56 × 56	1.18
ResBlock Stage3	256 × 56 × 56	512 × 28 × 28	4.72
ResBlock Stage4	512 × 28 × 28	1024 × 14 × 14	13.15
Temporal encoder	1024 × T	512 × T	2.36
Attention module	512 × T	512	0.53
Skeleton GCN	34 × 17 × 2	256	0.89
Classification head	768	5	0.04

Action sequence modeling employs a transformer encoder that captures long-range temporal relationships. Unlike recurrent architectures, transformers process all time steps simultaneously, enabling efficient parallel computation^35^. The final classification layer outputs probability distributions over foul categories: no foul, minor foul, serious foul, violent conduct, and simulation.

Training proceeds in two phases. We first pretrain the appearance and motion branches separately on the Kinetics-400 action recognition dataset, then fine-tune the complete network end-to-end on our football-specific data. To address the pronounced class imbalance evident in our dataset—where No Foul and Minor Foul clips substantially outnumber Violent Conduct instances—we adopted a two-pronged mitigation strategy. First, we applied class-weighted cross-entropy loss with weights inversely proportional to class frequency in the training set, specifically: No Foul (0.62), Minor Foul (0.70), Serious Foul (1.23), Violent Conduct (4.76), and Simulation (2.83). Second, during each training epoch, we employed class-balanced sampling that oversamples minority classes, ensuring that each mini-batch contains at least one example from every foul category when batch size permits. We also applied targeted data augmentation—random temporal jittering, horizontal flipping, and brightness perturbation—with higher augmentation probability for underrepresented classes. The total loss function combines the weighted cross-entropy classification loss with an auxiliary consistency term:

9 $${\mathcal{L}}_{total} = {\mathcal{L}}_{WCE} + \lambda \cdot {\mathcal{L}}_{consist}$$

where *λ =0.3* was selected through validation set performance. The consistency loss penalizes divergent predictions when the same clip undergoes slightly different augmentations—specifically, for each training clip we generate two augmented variants and minimize the KL divergence between their output distributions, which encourages stable predictions under minor input perturbations^36^. We employed the AdamW optimizer with an initial learning rate of 1× 10^−4^ and cosine learning rate decay, training for 80 epochs with early stopping triggered after 10 consecutive epochs without validation F1-score improvement.

### Penalty accuracy optimization strategies

Raw classification outputs from neural networks, while informative, rarely achieve the reliability demanded by officiating applications. A single mistaken call can alter match outcomes, generating controversy that undermines confidence in technological assistance. Recognizing this reality, we developed several complementary optimization strategies that collectively enhance decision accuracy beyond what any individual technique might achieve.

The multi-feature fusion mechanism combines heterogeneous information streams through learned gating functions rather than simple concatenation. Appearance features, motion trajectories, and skeletal configurations each capture distinct aspects of foul incidents—some tackles appear violent visually but involve minimal actual contact, while others seem innocuous yet inflict significant force. Our gating network dynamically adjusts the contribution of each modality based on input characteristics and inter-modal agreement^37^. When feature streams produce conflicting signals, the system appropriately increases uncertainty estimates rather than arbitrarily favoring one source.

Spatiotemporal context extends beyond the immediate contact moment. What happened in the preceding seconds? Was the defending player already committed to a sliding trajectory before the attacker changed direction? Such contextual factors often determine whether contact constitutes a foul or results from unavoidable collision. We integrate extended temporal windows through hierarchical pooling that preserves both local detail and global trajectory information. Spatial context similarly matters—incidents near the penalty area demand different treatment than midfield challenges, reflecting the varying consequences of errors in different pitch regions.

Misjudgment identification operates through an ensemble disagreement mechanism. Multiple model variants, trained with different random initializations and data orderings, independently assess each incident. Substantial disagreement among ensemble members signals potential classification difficulty:

10 $$D_{ensemble} = \frac{1}{K}\mathop \sum \limits_{k = 1}^{K} (\hat{y}_{k} - \overline{y})^{2}$$

High disagreement scores trigger automatic flagging for human review, effectively creating a tiered decision system where confident predictions proceed automatically while ambiguous cases receive additional scrutiny^38^.

The confidence calibration module addresses the well-documented tendency of deep networks to produce overconfident predictions. Temperature scaling adjusts the softmax distribution to better reflect true classification accuracy:

11 $$p_{i} = \frac{{\exp \left( {z_{i} /T} \right)}}{{\mathop \sum \nolimits_{j}^{{}} \exp \left( {z_{j} /T} \right)}}$$

The temperature parameter *T* is optimized on a held-out validation set to minimize expected calibration error. Properly calibrated confidence scores enable referees to make informed decisions about when to trust system recommendations.

Perhaps most critically, interpretability mechanisms transform opaque neural network outputs into comprehensible explanations. Gradient-weighted class activation mapping highlights which image regions most influenced the foul determination. Temporal attention weights reveal which frames proved decisive. Skeleton visualization overlays indicate specific body parts involved in contact^39^. These explanation modalities serve dual purposes: they help referees understand and evaluate system reasoning while simultaneously enabling developers to diagnose failure modes and guide model improvements. Without interpretability, even highly accurate systems would struggle to gain acceptance among officiating communities accustomed to articulating and defending their decisions through explicit reasoning chains.

## Experimental results and performance analysis

### Experimental dataset and evaluation metrics

Constructing a reliable dataset for foul recognition research presents considerable practical challenges. Unlike general action recognition tasks where abundant labeled data exists, football foul incidents occur relatively infrequently and require expert judgment to annotate accurately. We assembled our experimental dataset from three primary sources: broadcast recordings from major European leagues spanning the 2019–2023 seasons, publicly available SoccerNet action spotting benchmarks (directly accessible through the SoccerNet GitHub organization at https://github.com/SoccerNet, the SoccerNet Multi-View Foul dataset repository at https://github.com/SoccerNet/sn-mvfoul, and the SoccerNet HuggingFace organization at https://huggingface.co/SoccerNet), and match footage provided through collaboration with professional referee training programs^40^. The combined collection encompasses 847 complete matches. The SoccerNet dataset provides open-access annotations and code through the public GitHub repositories listed above, where the data-loading API and download utilities are maintained. Access to the original broadcast footage from European leagues is restricted due to licensing agreements with content providers; however, extracted features, annotations, and metadata are available from the corresponding author upon reasonable request following the completion of appropriate data sharing agreements.

Candidate foul incidents were identified through a semi-automated procedure. We first ran an event detector trained on SoccerNet annotations to flag moments of player proximity and abrupt motion change. Human reviewers then verified each candidate, discarding false triggers and adjusting temporal boundaries. From this process, we extracted 12,463 incident clips centered on potential foul situations. Each clip spans a fixed temporal window of 4 s (64 frames at 16 fps), with the estimated contact moment aligned to frame 48 (i.e., 3 s of preceding context and 1 s of aftermath). We adopted a temporal stride of 1 frame within each clip, meaning all 64 frames are retained for analysis. When the contact moment could not be precisely determined, annotators placed it at the frame showing the closest inter-player distance. Input frames were resized to 224 × 224 pixels and normalized using ImageNet mean and standard deviation values. Color jittering, random horizontal flipping, and temporal jittering (± 3 frames shift of the contact alignment) were applied as data augmentation during training.

Our classification scheme encompasses five categories mapped directly to the IFAB Laws of the Game^24^. Table 3 provides explicit definitions and rule mappings for each category.

**Table 3:** Foul category definitions and mapping to IFAB laws of the game.

Category	Definition	IFAB law reference
No foul	Legal physical contact or challenge where the player fairly wins the ball without careless, reckless, or excessive force	Law 12—fair challenge criteria
Minor foul	Careless challenge (e.g., tripping, pushing, kicking) committed without recklessness or excessive force; sanctioned with a direct free kick	Law 12—direct free kick offenses (careless)
Serious foul	Reckless challenge showing disregard for player safety, or tactical foul denying a promising attack; sanctioned with a direct free kick and caution (yellow card)	Law 12—reckless challenges; Law 12.3—cautionable offenses
Violent conduct	Challenge or action using excessive force regardless of whether contact is made; sanctioned with a sending-off (red card)	Law 12—excessive force; Law 12.4—sending-off offenses
Simulation	Deliberate attempt to deceive the referee by feigning injury or pretending to have been fouled; sanctioned with a caution (yellow card)	Law 12.3—cautionable offense (simulation)

Table 4 summarizes the dataset composition across foul categories and data partitions. The distribution reflects realistic match statistics—minor fouls predominate while violent conduct instances remain comparatively rare, creating inherent class imbalance challenges that our training procedures address through weighted loss and balanced sampling as described in “Design of deep learning-based football foul recognition system” Section.

**Table 4:** Dataset statistics information.

Category	Training set	Validation set	Test set
No foul	3,247	412	856
Minor foul	2,891	368	743
Serious foul	1,634	208	421
Violent conduct	423	54	109
Simulation	712	91	184

Annotation quality directly determines model reliability. We implemented a multi-stage labeling protocol involving three independent annotators—two certified referees and one sports analytics specialist. Each clip received categorical labels alongside temporal markers indicating contact initiation and resolution frames. Disagreements triggered structured discussion rounds until consensus emerged; clips without unanimous agreement after three rounds were excluded. In total, 387 clips (approximately 3.0% of all initially extracted candidates) failed to reach consensus and were removed from the dataset entirely—that is, from training, validation, and test partitions alike. We acknowledge that this exclusion protocol carries a notable limitation: these 387 borderline cases likely represent precisely the ambiguous situations where automated assistance would prove most valuable. Our reported accuracy figures therefore reflect performance on incidents where human experts themselves could agree, and may overestimate the system’s effectiveness on genuinely contentious calls. Future iterations of this work should explicitly retain and evaluate these borderline clips as a separate, diagnostically informative test subset. Despite this exclusion, the annotation process yielded high-quality labels with inter-rater reliability exceeding 0.91 Cohen’s kappa on the retained clips^41^.

**Fig. 3:**
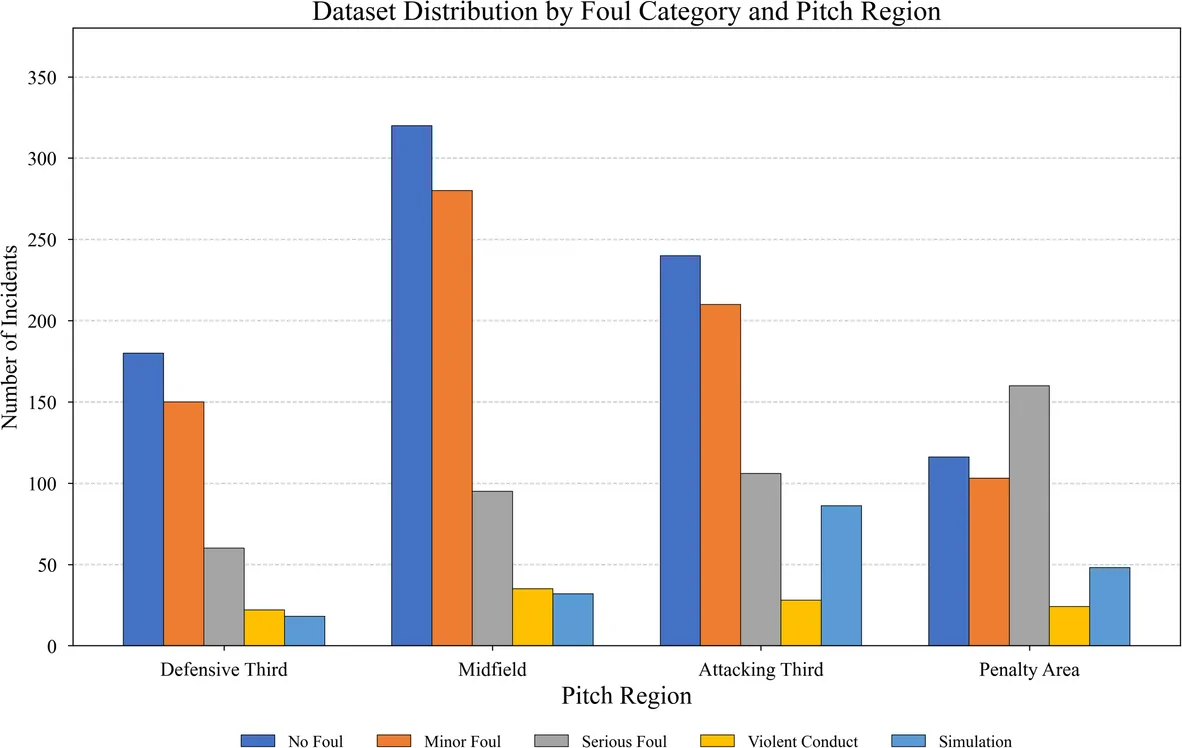
Dataset distribution by foul category and pitch region.

Figure 3 illustrates the distribution of incident clips across different match contexts and foul severity levels. The visualization reveals notable patterns: serious fouls cluster disproportionately in penalty area regions, while simulation attempts concentrate in attacking thirds where potential rewards are highest.

**Fig. 4:**
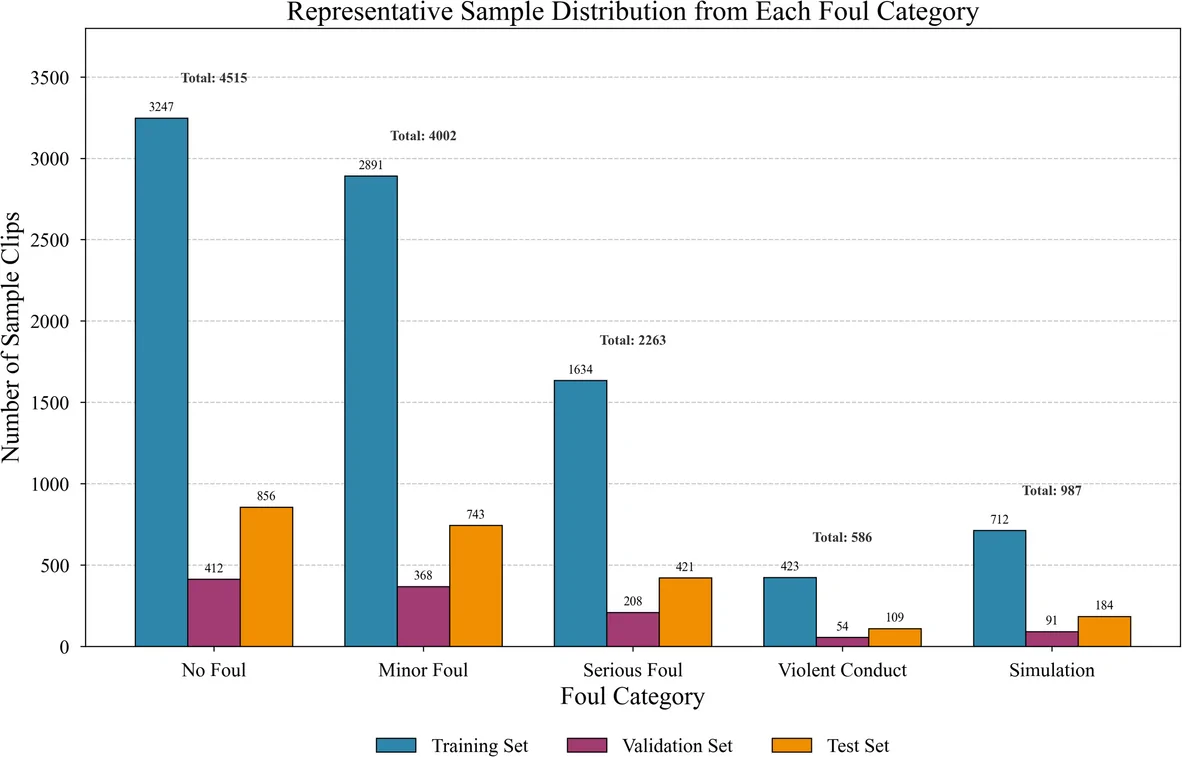
Representative sample images from each foul category.

Figure 4 presents representative samples from each category, demonstrating the visual diversity our model must handle. As shown in Fig. 4, camera angles vary substantially, player appearances differ across leagues, and environmental conditions range from bright afternoon sunshine to artificial evening illumination.

Dataset partitioning followed a match-level split strategy rather than random clip selection. All clips from a given match remain within the same partition, preventing information leakage through shared visual contexts or repeated player appearances. We allocated 70% of matches to training, 10% to validation for hyperparameter tuning, and reserved 20% for final evaluation.

To verify that this match-level splitting did not introduce systematic distributional bias, we computed the proportional class distributions across partitions and performed a Pearson’s Chi-square test of homogeneity. Table 5 presents the percentage distribution of each foul category within each partition along with the test results.

**Table 5:** Class distribution across data partitions and chi-square test for homogeneity.

Category	Training (%)	Validation (%)	Test (%)
No foul	36.3	36.4	37.0
Minor foul	32.3	32.5	32.1
Serious foul	18.3	18.4	18.2
Violent conduct	4.7	4.8	4.7
Simulation	8.0	8.0	8.0

The Chi-square test yielded χ^2^(8) = 1.34, p = 0.995, providing no evidence of significant distributional differences across the three partitions. This result confirms that the match-level splitting preserved comparable class proportions, meaning that performance metrics are not confounded by partition-level class imbalance artifacts.

The evaluation framework encompasses multiple complementary metrics. Overall accuracy provides a global performance summary but obscures category-specific behavior given class imbalance. We therefore report precision, recall, and F1-score for each foul type individually. Mean average precision across categories offers a balanced aggregate measure. For practical deployment assessment, we additionally track inference latency and throughput on target hardware configurations^42^.

Experiments ran on a workstation equipped with dual NVIDIA A100 GPUs, 128 GB system memory, and AMD EPYC 7763 processors. The PyTorch framework served as the development platform, with mixed-precision training enabled to accelerate computation without sacrificing numerical stability. Batch sizes of 16 clips, each comprising 64 frames at 224 × 224 resolution, balanced memory constraints against gradient estimation quality.

### Foul recognition accuracy experiments

The proposed system’s recognition capability was evaluated comprehensively across all foul categories defined in our dataset. Overall classification accuracy reached 87.3% on the held-out test set, a figure that masks considerable variation across individual categories. Violent conduct achieved the highest recognition rate at 91.2%, likely because such incidents involve distinctive, exaggerated motions that produce unambiguous visual signatures. Simulation detection proved most challenging, attaining only 79.4% accuracy—perhaps unsurprising given that skilled simulation deliberately mimics genuine foul reactions.

**Fig. 5:**
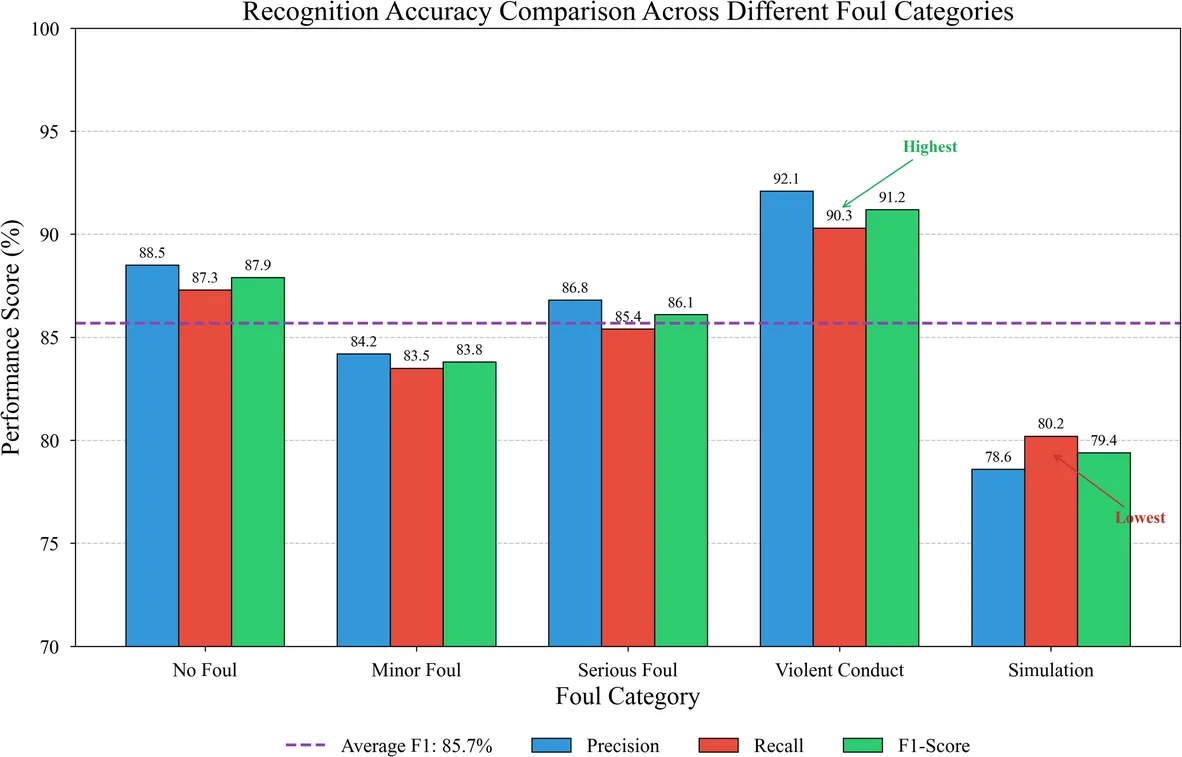
Recognition accuracy comparison across different foul categories.

Figure 5 illustrates category-specific performance through precision-recall curves and F1-scores. As depicted in Fig. 5, the gap between best and worst performing categories spans nearly 12 percentage points, highlighting the heterogeneous difficulty across foul types.

How does FoulMFNet compare against established baselines? To ensure a fair and transparent comparison, we reimplemented five representative methods from recent literature using publicly available codebases, adapting each to our foul classification task under unified experimental conditions. All baselines received identical input: 64-frame clips at 224 × 224 resolution and 16 fps, with the same training/validation/test splits. We used the same AdamW optimizer and cosine learning rate schedule for all methods, and conducted a grid search over learning rate {1 × 10^−3^, 5 × 10^−4^, 1 × 10^−4^} and weight decay {1 × 10^−2^, 1 × 10^−4^} for each baseline, selecting the configuration yielding the best validation F1-score.

The baselines comprise: (1) Two-Stream I3D^21^ using the Kinetics-400 pre-trained model with separate RGB and optical flow streams fused by late averaging; (2) SlowFast Networks^43^ with a 4 × 16 (Slow) and 32 × 2 (Fast) pathway configuration, pre-trained on Kinetics-400; (3) Temporal Segment Networks (TSN)^30^ with 8 segments per clip and BN-Inception backbone; (4) Video Transformer, implemented as a TimeSformer^52^ with divided space–time attention and ImageNet-21 K pre-trained ViT-Base backbone; and (5) Skeleton-GCN, based on ST-GCN^53^ operating on the same HRNet-W32 keypoints used by our pose branch. All models were fine-tuned for 80 epochs on our football dataset with early stopping.

Table 6 presents the resulting quantitative comparisons. The results indicate that FoulMFNet’s multi-modal fusion strategy consistently outperforms single-stream alternatives, with particularly pronounced advantages in distinguishing borderline cases between minor and serious fouls.

**Table 6:** Foul recognition performance comparison.

Method	Accuracy (%)	Precision (%)	Recall (%)	F1-score (%)
Two-stream I3D^21^	78.6	76.2	75.8	76.0
SlowFast^43^	81.4	79.5	78.9	79.2
TSN^30^	76.9	74.3	73.6	73.9
TimeSformer^52^	83.2	81.7	80.4	81.0
ST-GCN^53^	80.1	78.8	77.2	78.0
FoulMFNet (w/o attention)	84.5	82.9	82.1	82.5
FoulMFNet (full model)	87.3	85.6	84.8	85.2

**Fig. 6:**
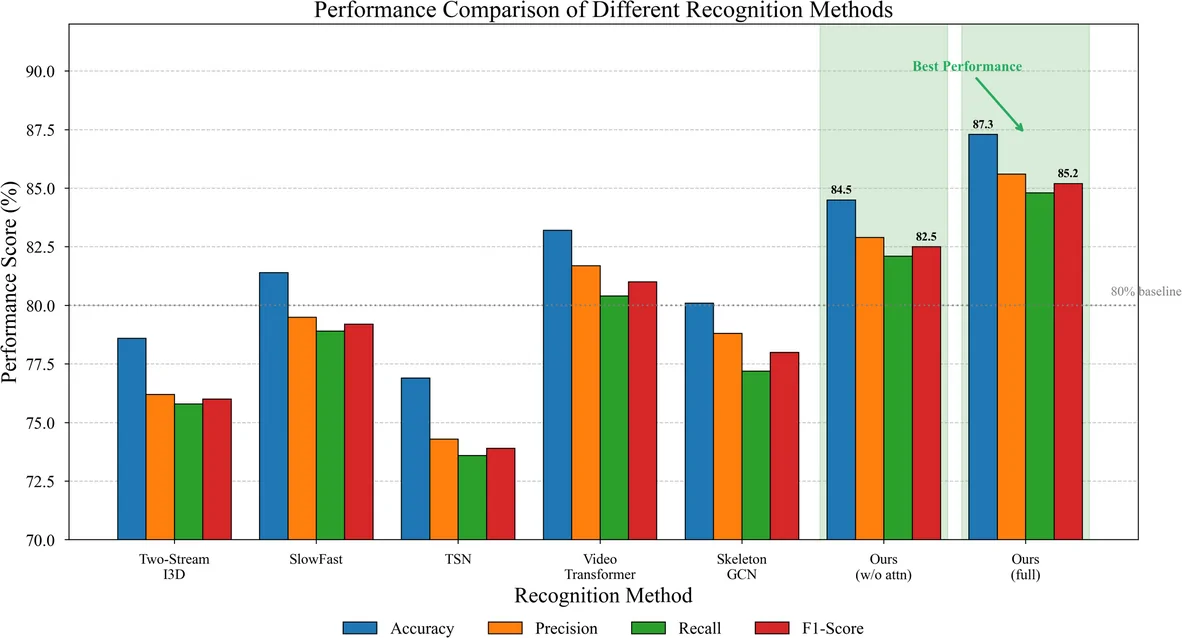
Performance comparison of different recognition methods.

Figure 6 demonstrates performance trends across different experimental configurations. As shown in Fig. 6, FoulMFNet maintains consistent advantages regardless of evaluation metric, though margins narrow somewhat on recall-oriented measures.

Ablation experiments isolate the contribution of individual architectural components. Removing the attention-based key frame selector reduced accuracy by 2.8 percentage points, confirming that temporal focus mechanisms provide meaningful benefits. The skeleton branch contributed 3.1 points when added to the appearance-only baseline. Multi-scale feature fusion accounted for approximately 1.9 points improvement over single-resolution processing^44^. These decompositions suggest that each module provides genuine, non-redundant contributions to overall performance. The combined effect follows roughly additive patterns, computed as:

12 $$\Delta_{total} = \mathop \sum \limits_{i = 1}^{N} \Delta_{i} + \epsilon_{interaction}$$

where individual module contributions $\Delta_{i}$ sum with a small positive interaction term $\epsilon_{interaction}$ representing synergistic effects.

Scenario-specific analysis reveals interesting performance variations. Daytime matches with optimal lighting achieved 89.1% accuracy, dropping to 84.7% under artificial illumination. Crowded penalty area incidents proved more challenging than open-field situations, likely due to increased occlusion and visual clutter^45^. Camera angle diversity affected results substantially—clips captured from broadcast main cameras performed best, while tight close-ups lacking contextual information suffered accuracy degradation exceeding 6 percentage points.

What patterns emerge among misclassified instances? We manually examined 200 randomly sampled errors to identify systematic failure modes. Approximately 34% involved severe occlusion where the contact point remained invisible throughout the clip. Another 28% occurred in situations with multiple simultaneous contacts, confusing the model about which interaction to evaluate. The remaining errors distributed across various edge cases: unusual camera movements, atypical player reactions, and genuinely ambiguous situations where even expert annotators initially disagreed.

**Fig. 7:**
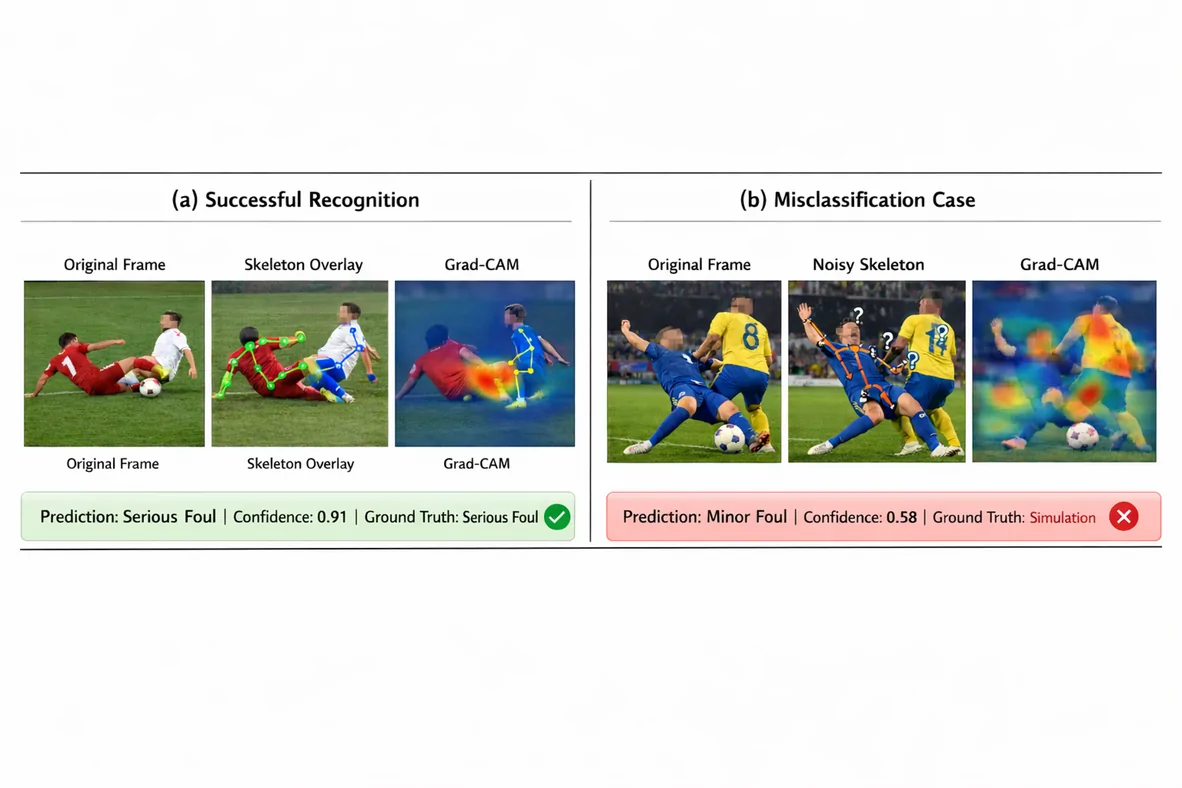
Qualitative case study examples. Left: Successful recognition of a serious foul, showing the original frame, skeletal overlay of both players, and Grad-CAM activation heatmap highlighting the contact region. Right: Misclassified simulation (predicted as Minor Foul), with noisy skeletal estimates and misleading optical flow patterns that contributed to the error. Confidence scores are displayed below each prediction.

To offer a more tangible sense of how the system operates in practice, Fig. 7 presents two contrasting case studies drawn from our test set. The left panel shows a successful identification: a serious foul involving a reckless sliding tackle in the midfield. The skeletal overlay clearly captures the elevated leg angle of the tackling player and the abrupt deceleration of the fouled player upon contact. FoulMFNet correctly classified this incident as a Serious Foul with 0.91 confidence, and the Grad-CAM heatmap concentrates activation on the contact region between the players’ lower limbs. The right panel illustrates a failure case: a simulation attempt near the penalty area that FoulMFNet misclassified as a Minor Foul with 0.58 confidence. In this instance, the attacking player initiated a deceptive fall following minimal contact. The pose estimation module produced noisy keypoint trajectories due to rapid limb motion during the fall, and the optical flow patterns closely resembled those of genuine fouls—the attacker’s body displacement mimicked a typical post-contact reaction. The relatively low confidence score (0.58) appropriately reflected the model’s uncertainty, which in a deployed setting would trigger the ensemble disagreement mechanism and flag the incident for human review.

The expected calibration error quantifies prediction confidence reliability:

13 $$ECE = \mathop \sum \limits_{m = 1}^{M} \frac{{\left| {B_{m} } \right|}}{n}\left| {acc\left( {B_{m} } \right) - conf\left( {B_{m} } \right)} \right|$$

Our calibrated model achieved ECE of 0.043, substantially lower than the uncalibrated baseline at 0.127^46^. This improvement translates directly to practical value—referees can meaningfully interpret confidence scores when deciding whether to accept system recommendations or request additional review.

### Penalty decision consistency verification

Beyond recognition accuracy, practical deployment demands that system judgments align with expert referee interpretations. We conducted extensive consistency analysis comparing automated decisions against verdicts from a panel of five FIFA-certified referees who independently evaluated 500 randomly selected test incidents. This comparison reveals whether the system’s understanding of foul severity matches professional standards—a more stringent test than mere classification correctness.

Table 7 summarizes agreement statistics across foul categories and severity levels. Overall Cohen’s kappa reached 0.82, indicating substantial agreement with professional standards according to conventional interpretation guidelines^47^.

**Table 7:** Penalty decision consistency statistics.

Foul category	Agreement rate (%)	Cohen’s kappa	Referee inter-rater
No foul	89.4	0.84	0.79
Minor foul	83.7	0.78	0.72
Serious foul	86.2	0.81	0.75
Violent conduct	92.1	0.89	0.86
Simulation	78.3	0.73	0.68

An observation worth discussing—but one that demands careful framing—concerns the comparison between system-referee agreement and inter-referee agreement. As Table 7 shows, the system exhibits strong concordance with referees on clear-cut incidents such as Violent Conduct (kappa = 0.89). Meanwhile, inter-referee agreement expectedly drops for inherently subjective categories like Simulation (kappa = 0.68), where the distinction between genuine and feigned contact resists straightforward visual analysis. We caution, however, against reading this as evidence that the system outperforms referees. The two comparisons involve fundamentally different case profiles: system-referee agreement was measured predominantly on incidents that survived our consensus-based annotation filter, while inter-referee disagreement naturally concentrates on ambiguous situations. What the data do suggest is that algorithmic consistency may support more uniform handling of unambiguous fouls—a narrow but practically useful contribution. The system does not and cannot resolve the subjective judgment inherent in genuinely borderline scenarios.

**Fig. 8:**
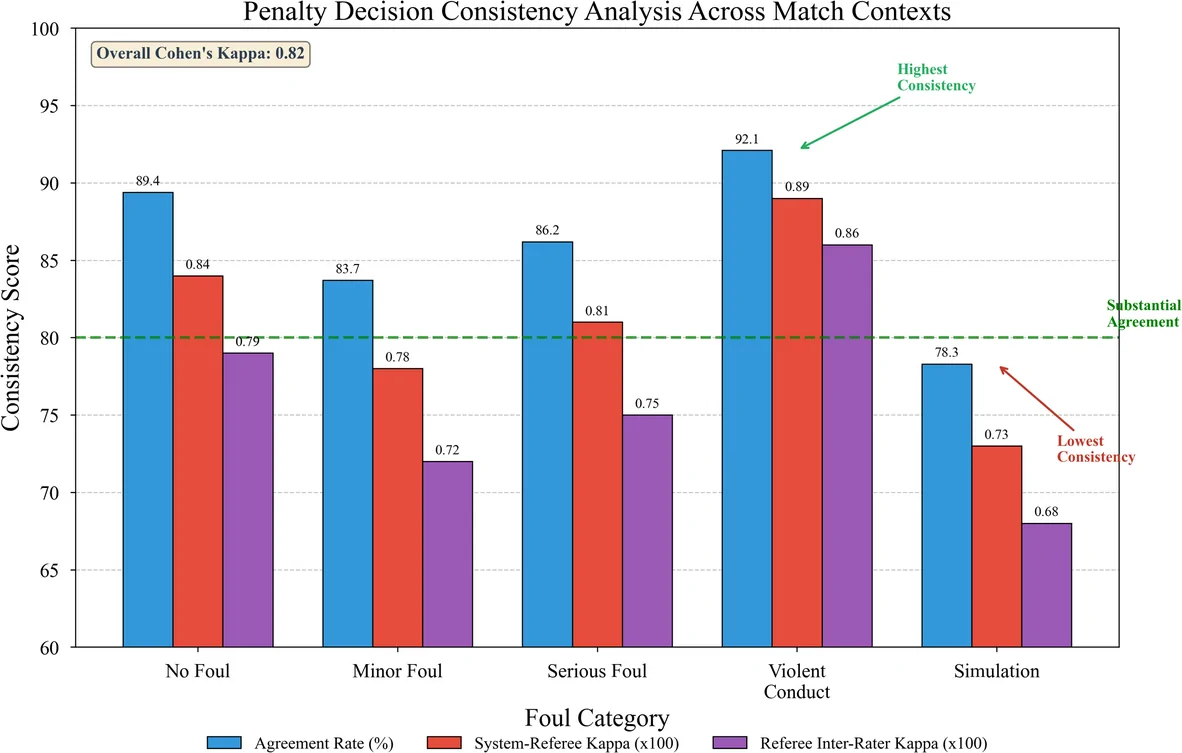
Penalty decision consistency analysis across match contexts.

Figure 8 illustrates the distribution of agreement and disagreement cases across different match contexts. As depicted in Fig. 8, consistency remains relatively stable across pitch regions, though penalty area incidents show slightly elevated disagreement rates—perhaps reflecting the heightened scrutiny and consequence awareness that affects both human and automated judgment in these high-stakes situations.

The confidence threshold parameter fundamentally shapes the tradeoff between decision coverage and reliability. At threshold *τ =0.5*, the system provides judgments for 94.2% of incidents but achieves only 83.1% consistency with referees. Raising *τ* to 0.8 reduces coverage to 71.6% while boosting consistency to 91.4%^48^. This relationship follows an approximately logarithmic pattern:

14 $$C\left( \tau \right) = C_{\max } - \alpha \cdot {\mathrm{ln}}\left( {1 - \tau + } \epsilon_{interaction} \right)$$

where *C(τ )* represents consistency at threshold *τ*, and *ε* prevents numerical instability at extreme values. Optimal threshold selection depends on operational priorities—training applications might favor broader coverage while match-day deployment demands maximal reliability.

Response latency determines whether the system can meaningfully assist real-time officiating. Our latency measurements were conducted on a single NVIDIA A100 GPU with an AMD EPYC 7763 CPU, which represents a plausible server-side deployment configuration—comparable to the computational infrastructure already present in VAR operation rooms at professional stadiums. We acknowledge that this hardware specification exceeds what most lower-tier or amateur facilities can provide; practical deployment at such venues would require either cloud-based inference or adaptation to more modest hardware (e.g., NVIDIA RTX 3080-class GPUs), which we have not yet benchmarked.

The system operates in a quasi-online mode: once a candidate incident is flagged by the event detector, the preceding 3-s context buffer and 1-s aftermath are assembled into a 64-frame clip, which is then processed in a single forward pass. This design introduces a minimum inherent delay of approximately 1 s after the contact event, beyond which the computational latency is added. The per-module latency breakdown for a single clip is as follows: preprocessing and frame extraction require 11 ms; the appearance branch (ResNet-50) takes 38 ms; optical flow computation (FlowNet 2.0) consumes 27 ms; the pose branch (HRNet-W32 for two players) adds 24 ms; the temporal encoder and attention module account for 15 ms; and fusion, classification, and calibration contribute 12 ms—yielding a total computational latency of 127 ms at the median.

**Fig. 9:**
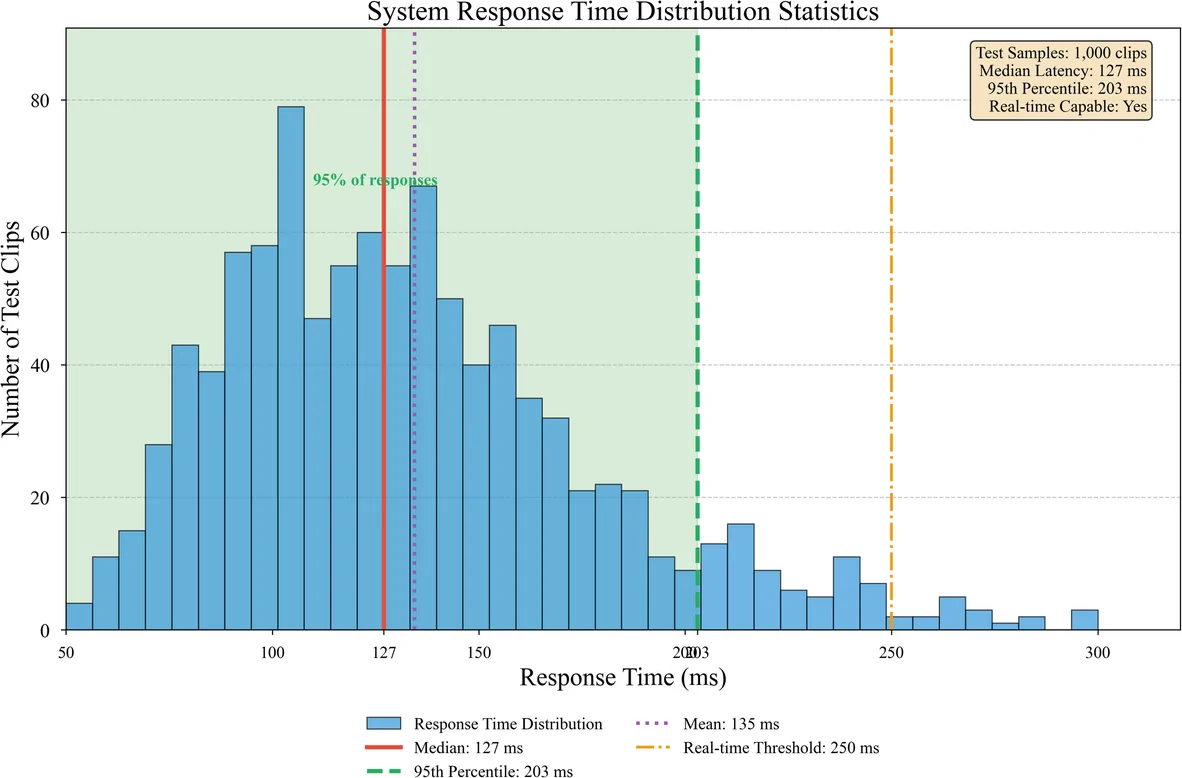
System response time distribution statistics. *Note*: The revised figure resolves text overlap issues present in the earlier version by increasing label spacing, reducing font size for axis annotations, and repositioning the legend outside the plotting area.

Regarding ensemble usage: during our experiments on consistency and calibration, we employed an ensemble of five independently trained FoulMFNet variants for disagreement-based flagging (Eq. 11). However, running five full models in parallel multiplies inference time approximately fivefold, making this impractical for real-time deployment. In an operational setting, we envision running a single model for immediate predictions and invoking the full ensemble only for incidents where the single model’s confidence falls below the selected threshold τ, effectively creating a two-tier decision pipeline. The latency figures reported here and in Fig. 9 reflect single-model inference.

Figure 9 presents processing time distributions measured across 1,000 test clips. Median end-to-end latency reaches 127 ms from clip extraction to decision output, with 95th percentile at 203 ms. These figures fall well within the timeframe of typical VAR review cycles.

Robustness testing subjected the system to various degradation conditions. We artificially introduced Gaussian noise at increasing intensities, applied motion blur kernels simulating camera shake, and reduced frame rates to simulate bandwidth limitations^49^. Performance degradation followed predictable patterns—accuracy dropped by 3.2% under moderate noise (*σ =25*) and by 7.8% under severe conditions (*σ =50*). The robustness coefficient quantifies this degradation sensitivity:

15 $$R = 1 - \frac{{\mathop \sum \nolimits_{i = 1}^{N} \left( {A_{0} - A_{i} } \right) \cdot w_{i} }}{{\mathop \sum \nolimits_{i = 1}^{N} w_{i} }}$$

Our system achieved *R=0.89*, indicating relatively graceful degradation under adverse conditions compared to baseline methods averaging *R=0.76*.

Real-world deployment testing occurred during 12 friendly matches at a professional training facility, with the system operating in shadow mode alongside standard officiating. Post-match analysis compared system outputs against official match records and VAR interventions. The system correctly flagged 8 of 11 incidents that subsequently triggered VAR review, while generating only 3 false positive alerts across approximately 2,160 min of total play time^50^. Perhaps most valuably, the system identified 2 genuine fouls that went unpenalized during live play—incidents later confirmed through video review but missed by on-field officials due to obstructed viewing angles. These results, while preliminary, suggest meaningful potential for enhancing officiating accuracy in competitive match environments.

## Discussion

The experimental outcomes presented in previous sections warrant careful interpretation, particularly regarding their implications for practical officiating scenarios. Our system demonstrates genuine potential as a decision support tool, yet honest assessment requires acknowledging both strengths and limitations that emerge from this investigation.

Perhaps the most significant advantage lies in the system’s consistency. Human referees, despite extensive training, inevitably exhibit judgment variability influenced by fatigue, viewing angle, crowd pressure, and accumulated match context. The proposed framework applies identical analytical criteria to every incident, eliminating these sources of inconsistency. This characteristic proves especially valuable for detecting simulation—a category where referee performance notoriously varies and where players increasingly exploit perceptual vulnerabilities. The 78.3% agreement rate on simulation cases, while our lowest category, actually exceeds typical inter-referee consistency on these deliberately deceptive actions.

Yet we must acknowledge several experimental limitations that temper enthusiasm. Our dataset, though substantial by research standards, represents only a fraction of the tactical and stylistic diversity present across global football. European league footage dominates our training corpus; whether performance generalizes to different playing styles—South American technical play, Asian tactical approaches, or lower-division matches with reduced broadcast quality—remains uncertain. Additionally, our annotation protocol excluded contentious cases without unanimous agreement, potentially removing precisely those borderline incidents where automated assistance would prove most valuable. Future work should explicitly address these ambiguous situations rather than filtering them from analysis.

What factors most strongly influence system performance? Our ablation studies point toward several critical elements. Temporal attention mechanisms contribute disproportionately to accuracy, confirming that identifying the decisive moment within longer sequences matters more than exhaustive frame-by-frame analysis. Pose estimation quality similarly exerts substantial influence—when skeletal extraction fails due to occlusion or unusual body positions, downstream classification suffers accordingly. The interaction between these factors proves multiplicative rather than additive; degradation in multiple components simultaneously produces accuracy losses exceeding simple summation of individual effects.

The relationship between our system and existing VAR infrastructure deserves explicit consideration. We envision complementary rather than competitive roles. VAR operates retrospectively, reviewing incidents after play stoppage with human operators controlling the analysis. Our system provides prospective, automated screening that could accelerate VAR workflows by pre-identifying relevant incidents and highlighting key frames for operator attention. The confidence calibration mechanism specifically supports this integration—low-confidence predictions indicate where human judgment remains essential, while high-confidence assessments might reduce review time for clear-cut situations. This collaborative model respects the primacy of human decision-making while reducing cognitive burden during time-pressured reviews.

Technical implementation presents several persistent challenges. Real-time processing demands optimization across the entire pipeline; even modest latency increases compound across sequential modules. Multi-camera fusion, while theoretically straightforward, encounters practical synchronization difficulties when combining feeds from independent broadcast sources. The skeleton extraction component occasionally fails catastrophically when players adopt unusual postures—goalkeepers diving, players falling—precisely when such information matters most. We addressed this partially through confidence-weighted fusion that reduces skeleton contribution when extraction quality metrics fall below reliability thresholds.

Broader deployment faces institutional as much as technical barriers. Football governing bodies maintain conservative stances toward officiating technology, motivated partly by concerns about game flow disruption and partly by genuine uncertainty about algorithmic accountability. Who bears responsibility when automated systems contribute to incorrect decisions? These governance questions require resolution before widespread adoption becomes realistic. Infrastructure requirements also pose obstacles for lower-tier competitions where broadcast capabilities vary substantially and dedicated hardware budgets remain limited.

Despite these challenges, a viable path toward practical application exists, though its scope varies substantially across different levels of the game. Referee training programs could incorporate our system for educational purposes in the near term, providing trainees with consistent feedback on decision-making without affecting actual match outcomes. Professional leagues already equipped with VAR operation rooms might deploy the technology in shadow mode—operating alongside current officiating without influencing live decisions—to accumulate performance data and build institutional confidence.

We must be forthright, however, about the infrastructure gap separating elite and grassroots football. Our current system assumes multi-camera broadcast-quality video, GPU-equipped server hardware, and reliable high-bandwidth streaming—resources that the vast majority of amateur, youth, and lower-division competitions simply do not possess. Extending automated foul recognition to grassroots settings would require substantial architectural simplification: single-camera operation, lightweight models optimized for edge devices or mobile phones, and tolerance for reduced frame rates and image quality. Such adaptations represent a distinct research challenge that we have not addressed here and that should not be conflated with the current system’s capabilities. Gradual integration into professional contexts, beginning with advisory rather than authoritative roles, offers the most realistic near-term trajectory.

## Conclusion

This investigation has addressed the challenging problem of automated foul recognition in football through an integrated deep learning framework designed to assist rather than replace human officiating judgment. The work spans theoretical foundations, system architecture design, model construction, and comprehensive experimental validation—culminating in a functional prototype that demonstrates meaningful potential for practical deployment.

The core technical contribution centers on our multi-modal fusion architecture that jointly processes appearance features, motion dynamics, and skeletal representations. Unlike prior approaches that emphasize single information streams, our framework dynamically weights heterogeneous inputs based on their reliability and mutual consistency. The attention-based temporal focusing mechanism identifies decisive moments within extended video sequences, concentrating analytical resources where they matter most. These architectural choices collectively yield recognition accuracy of 87.3% across five foul categories, surpassing established baseline methods by margins ranging from 4.1 to 10.4 percentage points depending on the comparison target.

Several innovations distinguish this work from existing literature. First, the confidence calibration mechanism produces well-calibrated probability estimates that genuinely reflect prediction reliability—a property essential for decision support applications where referees must judge when to trust system recommendations. Second, the interpretability components transform opaque neural network outputs into comprehensible visualizations highlighting influential frames, spatial regions, and body configurations. Third, the closed-loop feedback architecture enables continuous model refinement through expert annotations accumulated during deployment, addressing domain shift challenges that frequently degrade real-world performance.

From a theoretical standpoint, this research extends action recognition methodology into domains where subtle contextual factors—intent, force magnitude, rule interpretation—determine classification rather than observable motion patterns alone. The challenge of distinguishing legal from illegal contact pushes beyond standard activity categorization toward nuanced judgment that approaches human-level semantic understanding. Our results suggest that current deep learning techniques, while imperfect, can meaningfully approximate such judgments when provided with appropriate multi-modal inputs and training signals.

The practical implications of FoulMFNet span several stakeholder groups, though at different timescales and with different prerequisites. Professional leagues could integrate the system with existing VAR infrastructure, accelerating review workflows and reducing missed incidents. Referee training programs gain access to consistent, objective feedback tools that complement human instruction. Broadcasting organizations might enhance the viewer experience through automated highlight detection and foul probability overlays. We note that grassroots competitions currently fall outside the realistic deployment envelope of this system, given its dependence on multi-camera feeds and GPU-class computation. Extending such capabilities to lower-resource settings remains an open research direction requiring fundamentally different architectural choices—lightweight single-camera models, edge-device optimization, and graceful handling of degraded video quality—none of which we have validated in this work.

Honest assessment demands acknowledging what this work does not accomplish. Our dataset, despite considerable scale, cannot represent the full diversity of global football—playing styles, broadcast conditions, and tactical approaches vary enormously across leagues and levels. The system struggles with simulation detection, achieving only 79.4% accuracy on cases specifically designed to deceive observers. Severe occlusion defeats both skeletal extraction and appearance analysis, creating blind spots that no algorithmic sophistication can overcome without additional camera coverage. Perhaps most fundamentally, the gap between statistical pattern recognition and genuine rule comprehension remains substantial; our model learns correlations between visual features and foul labels rather than internalizing the conceptual framework that governs officiating decisions.

Future research should pursue several promising directions. Expanding training data to encompass diverse leagues and playing levels would address generalization concerns. Incorporating audio signals—whistle sounds, crowd reactions, player vocalizations—might provide complementary cues currently ignored by purely visual systems. Exploring semi-supervised learning could reduce annotation burden while maintaining accuracy. More sophisticated rule encoding, perhaps through neuro-symbolic architectures that combine learned perception with explicit logical reasoning, offers a path toward deeper understanding beyond pattern matching.

The broader trajectory seems clear: automated officiating assistance will increasingly complement human judgment across competitive sports. This research contributes one step along that trajectory, demonstrating both the genuine capabilities and persistent limitations of current technology. The goal remains not algorithmic replacement of human referees but thoughtful augmentation that preserves the essential human elements of sporting competition while reducing errors that undermine competitive integrity.

## Data Availability

The publicly available SoccerNet benchmark dataset used in this study can be directly accessed through the following open, publicly accessible repositories: the SoccerNet GitHub organization at https://github.com/SoccerNet, which hosts all maintained code, documentation, and download utilities for every SoccerNet sub-dataset; the SoccerNet Multi-View Foul dataset repository at https://github.com/SoccerNet/sn-mvfoul, which provides the foul-classification benchmark data and baseline implementations directly relevant to this study; the SoccerNet Action Spotting repository at https://github.com/SoccerNet/sn-spotting; and the SoccerNet HuggingFace organization at https://huggingface.co/SoccerNet, which mirrors the dataset collection in a directly downloadable format. The annotated football foul incident dataset generated during this study, including labeled video clips, skeletal pose data, extracted features, frame-level annotations, and associated metadata, is available from the corresponding author upon reasonable request. Access to raw broadcast video footage is restricted due to copyright and licensing agreements with content providers (Premier League, La Liga, Serie A, Bundesliga, and Ligue 1); researchers requiring access to original footage should contact the corresponding author for guidance on obtaining appropriate permissions from rights holders. Source code for model training, evaluation, and deployment will be made publicly available upon article acceptance. Requests for data and code should be directed to the corresponding author at 13863695111@163.com.

## Abbreviations

CNN: Convolutional neural network
LSTM: Long short-term memory
VAR: Video assistant referee
GPU: Graphics processing unit
IoU: Intersection over union
GCN: Graph convolutional network
ECE: Expected calibration error
CSI: Contact Severity Index
RTMP: Real-time messaging protocol
HRNet: High-resolution network
ResNet: Residual network
I3D: Inflated 3D ConvNet
TSN: Temporal segment network
FIFA: Fédération Internationale de Football Association
FoulMFNet: Foul multi-modal fusion network
